# Cross-Sectional Study of Sexual Activity and Satisfaction Among Older Adult's ≥60 Years of Age

**DOI:** 10.1016/j.esxm.2020.100316

**Published:** 2021-03-03

**Authors:** Magnus Stentagg, Lisa Skär, Johan Sanmartin Berglund, Terese Lindberg

**Affiliations:** 1 Department of Care Sciences, Malmö University, Malmö, Sweden; 2 Department of Health, Blekinge Institute of Technology, Karlskrona, Sweden

**Keywords:** Elderly, Older Adults, Sexual Activity, Sex, Sexual Health, Sexual Satisfaction, SNAC

## Abstract

**Introduction:**

Despite the rapidly increasing population of older adults, little is currently known about sexual activity and sexual satisfaction among the oldest people.

**Aim:**

The present study aimed to investigate sexual activity and sexual satisfaction among people of ≥60 years of age. We also examined whether sexual activity and sexual satisfaction were influenced by age, gender, cohabiting, socioeconomic factors, education, functional ability, and self-reported health.

**Methods:**

We performed a descriptive analysis of self-stated sexual activity and sexual satisfaction among 1680 participants who were 60 years and older from the Swedish National Study on Aging and Care. Chi-square tests and logistic regression were used to analyze relationships between factors.

**Main Outcome Measure:**

Sexual activity and sexual satisfaction.

**Results:**

Among participants aged ≥90 years, about 10% were sexually active. Within the total study population, 46% (654/1680) were sexually active. Overall, sexually activity was more commonly reported by men (55%) than women (40%). However, men in all age cohorts reported sexual dissatisfaction more commonly than women. In the total sample, 24% (246/1680) reported dissatisfaction with their sex life. Sexual activity and sexual satisfaction were positively associated with self-reported health and cohabitation.

**Conclusion:**

The present results suggest that sexual activity is present throughout life. For persons older than 90 years, about 10% of participants were sexually active, regardless of gender. Every third man reported dissatisfaction with his sex life. Women were more satisfied with their sex lives than men, and this difference varies more widely among age cohorts. These findings confirm that it is important that health professional take sexuality into account during caring encounters with older persons.

**M Stentagg, L Skär, JS Berglund, et al. Cross-Sectional Study of Sexual Activity and Satisfaction Among Older Adult's ≥60 Years of Age. Sex Med 2021;9:100316.**

## Introduction

Limited information is available regarding older people's sexual activity and sexual satisfaction, and how these factors relate to health and quality of life.^1^^,^^2^ There is also a lack of studies including the oldest old. Older individuals are often viewed as asexual, and research relating to human sexuality in this population is commonly presented from a biomedical approach, highlighting sexual health problems related to aging.^3^ Few studies have actually investigated the levels of sexual activity and sexual satisfaction among people aged 60 years or older.^4^ Previous studies show decreasing sexual activity with age^5^, ^6^, ^7^, ^8^, ^9^ but this does not necessarily indicate that sexual satisfaction also declines. Sexual satisfaction seems to be affected by health rather than by age.^10^ Elderly people who estimate their health as good or very good have a much more positive attitude toward sex and sexuality.^4^^,^^5^ One study in a large group of older people found that one or more sexual dysfunctions are directly linked to reduced sexual satisfaction.^11^ Sexual activity and sexual satisfaction are considered an indicator for measuring people's ability to manage their activities of daily living.^12^ Among individuals aged 75–85 years, Lindau et al^13^ found that approximately half of participants were satisfied with their sex lives, with good sex life quality reported by 50.9% of women and 70.8% of men. Gott and Hinchliff^3^ and Penhollow et al^14^ report that sexual satisfaction is strongly associated with having an intimate partner relationship. Other studies report that good physical health, the availability of a partner, and a regular and stable pattern of sexual activity earlier in life predict the maintenance of sexual activity in old age.^15^

Many older people living in a partner relationship see sexual activity as a natural and important part of the relationship.^1^ Socioeconomic status reportedly has an impact on self-reported health; thus, it is reasonable to assume that it also affects the sexual interest. However, few sexology research studies have investigated this possibility, particularly in the perspective of an older population. Younger cohorts are more frequently studied, and a Danish study of 40-year-old women showed that women with higher socioeconomic backgrounds had sex more often and enjoyed it to a greater extent than those with lower socioeconomic backgrounds.^16^ Sexual satisfaction is considered an important factor that can positively affect health. However, many previous studies ignore the positive side and life-enhancing functions of sexuality. Older individuals are often viewed as asexual and studies with the oldest population is lacking therefore additional knowledge of sexual activity and sexual satisfaction among older people and the associations with demographic factors, health, and functional ability, may help health care professionals improve quality of life in this population.

The aim of the present study was therefore to investigate sexual activity and sexual satisfaction among people aged ≥60 years. It was hypothesized that sexual activity and sexual satisfaction were affected by age, sex, cohabiting, socioeconomic factors, education, functional ability, and self-reported health.

## Methods

### Sample

Data were collected from one of the 4 regions included in the Swedish National Study on Aging and Care (SNAC), a longitudinal national study of older adults living in Sweden that started in 2001. Lagergren et al^17^ provide a detailed description of the SNAC study. The present study used data collected from 2001 to 2010 at SNAC-Blekinge (SNAC-B) in the Karlskrona municipality, a medium-sized Swedish town with rural surroundings that is situated in southeast Sweden. The reason that data are only taken from this region was that questions about sex are not in the other regions. All participants gave informed consent for participation in SNAC.

The presented sample comprised 721 men and 959 women (n = 1680) living in the Karlskrona municipality, who were randomly selected and divided into 10 age cohorts. The participants’ ages ranged from 60 to 96 years, with the following distribution: 24%, 60 years of age; 12%, 66 years; 11%, 72 years, 10%, 78 years, 13%, 81 years; 12%, 84 years, 9%, 87 years, 6%, 90 years, 2%, 93 years, and 1% were 96 years of age. The median age in the sample was 78 years. Before the analyses were performed the cohorts were divided into 4 quadrants 60-66, 72,78, 81-87, and ≥ 90.

The SNAC-B study was approved by the Regional Ethical Review Board in Lund (LU 605-00, LU 744-00).

### Sexual Activity and Sexual Satisfaction

Sexual activity and sexual satisfaction were assessed based on 2 questions. The first was “Have you been sexually active in the last year?” and was answered with one of the following selections: no 53.7%, yes sometimes (occasionally) 18.5%, yes about once a month (regularly) 11.1%, yes about once a week or every day (often) 16.6%. As the purpose was only to find out if the participant had been active or not, the answers were combined which after merging gave 2 equal groups. The second question was “Are you sexually satisfied?” and was answered by yes or no.

### Demographics

The covariates included in the study were age, gender, education, cohabitation, and household economy. For the demographic analyses (Tables 1 and 2), age was recoded as a categorical variable with 4 age cohorts: 60–66, 72–79, 80–89, and 90 years and older. In the logistic regression (Tables 3 and 4), age was recoded into quartile in the range of 60-65, 66-71, 72-80, and 81 years and older. Education was categorized into 3 groups based on the previous Swedish education system, as was appropriate for the age groups in this study. The 3 groups were participants with only an elementary school education (low), those who had finished secondary school (mid), and those who held a university degree (high). Household economy was categorized based on whether the person had available savings, which was assessed using the cash margin question from the Swedish Statistics Survey on Income and Living Conditions. Participants answered the question “Within a week, are your assets and financial resources sufficient to meet emergencies of 14,000 Swedish crowns?” (14,000 Swedish crowns was used in the question from 2001 until 2006, and this was changed to 15,000 Swedish crowns from 2007). The response options were yes and no.

**Table 1:** Self-stated sexual activity of 60- to 96-year-old participants from the SNAC-B sample, in accordance with demographic characteristics, both in total and stratified by sex

Characteristic	Not sexually active n (%)	Sexually active n (%)	*P*-value
Individuals	760 (54%)	654 (46%)	.001
Male	271 (45%)	334 (55%)	
Female	489 (60%)	320 (40%)	
Age cohort			
60–66 years	142 (27%)	387 (73%)	.001
Male	51 (20%)	199 (80%)	
Female	91 (33%)	188 (67%)	
72–78 years	154 (53%)	139 (47%)	.091
Male	58 (47%)	64 (53%)	
Female	96 (56%)	75 (44%)	
81–87 years	352 (75%)	115 (25%)	.000
Male	132 (66%)	67 (34%)	
Female	220 (82%)	48 (18%)	
≥90 years	112 (90%)	13 (10%)	.493
Male	30 (88%)	4 (12%)	
Female	82 (90%)	9 (10%)	
Cohabiting	299(47%)	338 (53%)	
Male	164 (47%)	183 (53%)	
Female	135 (47%)	155 (53%)	
Low education	577 (61%)	372 (39%)	
Male	206 (54%)	173 (46%)	
Female	371 (65%)	199 (35%)	
Mid education	37 (42%)	52 (58%)	
Male	14 (29%)	34 (71%)	
Female	23 (56%)	18 (44%)	
High education	93 (32%)	202(68%)	
Male	35 (24%)	111 (76%)	
Female	58 (39%)	91 (61%)	
PADL score	291 (72%)	113 (28%)	
Dependent			
Male	82 (65%)	44 (35%)	
Female	209 (75%)	69 (25%)	
Independent	455 (46%)	535 (54%)	
Male	186 (39%)	287 (61%)	
Female	269 (52%)	248 (48%)	
Low household economic status	135 (63%)	79 (37%)	
Male	36 (51%)	34 (49%)	
Female	99 (69%)	45 (31%)	
High household economic status	562 (51%)	541 (49%)	
Male	220 (44%)	281 (56%)	
Female	342 (67%)	260 (33%)	
Poor self-reported health	338 (62%)	205 (38%)	
Male	105 (54%)	91 (46%)	
Female	233 (67%)	114 (33%)	
Good self-reported health	297 (42%)	402 (58%)	
Male	132 (37%)	223 (63%)	
Female	165 (48%)	179 (52%)	

*P* values < 0.05 represent the significance level for differences in sexually active and not, using chi-square test.

PADL score = personal activities of daily life, including dressing, bathing, toilet use, personal appearance, continence, transfer, and feeding.

**Table 2:** Sexual satisfaction of 60- to 96-year-old participants from the SNAC-B sample, in accordance with demographic characteristics, both in total and stratified by sex

Characteristic	Not sexually satisfied n (%)	Sexually satisfied n (%)	*P*-value
Individuals	246 (23%)	803 (77%)	.009
Male	163 (31%)	356 (69%)	
Female	83 (16%)	447 (84%)	
Age cohort			
60–66 years	127 (25%)	382 (75%)	.127
Male	78 (31%)	170 (69%)	
Female	49 (19%)	212 (81%)	
72–78 years	56 (25%)	167 (75%)	.315
Male	37 (32%)	77 (68%)	
Female	19 (17%)	90 (83%)	
81–87 years	55 (21%)	213 (79%)	.026
Male	44 (31%)	99 (69%)	
Female	11 (9%)	114 (91%)	
≥90 years	8 (17%)	41 (83%)	.217
Male	4 (29%)	10 (71%)	
Female	4 (11%)	31 (89%)	
Cohabiting	131 (23%)	438 (77%)	
Male	103 (31%)	225 (69%)	
Female	28 (12%)	213 (88%)	
Low education	151 (21%)	559 (79%)	
Male	52 (18%)	241 (82%)	
Female	99 (24%)	318 (76%)	
Mid education	26 (32%)	56 (68%)	
Male	18 (36%)	32 (64%)	
Female	8 (25%)	24 (75%)	
High education	66 (28%)	171 (72%)	
Male	44 (36%)	78 (64%)	
Female	22 (19%)	93 (81%)	
PADL score	45 (20%)	176 (80%)	
Dependent			
Male	23 (30%)	54 (70%)	
Female	22 (15%)	122 (85%)	
Independent	197 (24%)	618 (76%)	
Male	139 (32%)	297 (68%)	
Female	58 (15%)	321 (85%)	
Low household economic status	36 (24%)	116 (76%)	
Male	20 (36%)	35 (64%)	
Female	16 (16%)	81 (84%)	
High household economic status	205 (23%)	674 (77%)	
Male	140 (31%)	315 (69%)	
Female	65 (15%)	359 (85%)	
Poor self-reported health	110 (27%)	296 (73%)	
Male	65 (39%)	103 (61%)	
Female	45 (19%)	193 (81%)	
Good self-reported health	122 (20%)	474 (80%)	
Male	94 (28%)	244 (72%)	
Female	28 (11%)	230 (89%)	

*P* values < 0.05 represent the significance level for differences by sex using chi-square test. PADL score = personal activities of daily life, including dressing, bathing, toilet use, personal appearance, continence, transfer, and feeding.

**Table 3:** Logistic regression (first and last step) of the relationship between sexual activity as a dependent variable and PADL, cohabitation, self-reported health, and age as covariates, among 60- to 96-year-old participants from the SNAC-B sample (n = 987)

Step 1					
Dependent variable	Independent variable	B	*P*-value	OR	95% CI
Sexual activity	Sex (male)	0.313	.031	1.367	1.030-1.815
	Economic	−0.141	.464	0.868	0.595-1.267
	Cohabitation	0.835	.000	2.306	1.702-3.123
	PADL	0.326	.231	1.385	0.813-2.360
	Age 66-71	−0.533	.004	0.587	0.409-0.841
	Age 72-80	−1.585	.000	0.205	0.139-0.303
	Age ≥81	−2.157	.000	0.116	0.075-0.179
	Education (mid)	0.248	.134	1.282	0.927-1.773
	Education (high)	0.328	.106	1.388	0.933-2.064
	Self-reported health	0.333	.038	1.396	1.018-1.913
Step 5					
Sexual activity	Sex (male)	0.350	.014	1.419	1.073-1.878
	Cohabitation	0.837	.000	2.309	1.713-3.113
	Age 66-71	−0.583	.001	0.558	0.392-0.795
	Age 72-80	−1.685	.000	0.185	0.127-0.271
	Age ≥81	−2.287	.000	0.102	0.067-0.155
	Self-reported health	0.354	.027	1.425	1.041-1.950

OR = odds ratio; CI = confidence interval.

**Table 4:** Logistic regression model (first and last step) of the relationship between sexual satisfaction as a dependent variable and sex (male), self-reported health, and age as covariates among 60- to 96-year-old participants from the SNAC-B sample (n = 806)

Step 1					
Dependent variable	Independent variable	B	P-value.	OR	95% CI
Sexual satisfaction	Sex (male)	−0.586	.001	0.557	0.398-0.780
	Economic	0.283	.206	1.327	0.856-2.059
	Cohabitation	0.399	.035	1.490	1.028-2.159
	PADL	0.015	.965	1.015	0.525-1.962
	Age 66-71	0.147	.493	1.159	0.761-1.765
	Age 72-80	0.060	.797	1.062	0.673-1.675
	Age ≥81	0.711	.010	2.037	1.183-3.507
	Education (mid)	−0.086	.656	0.918	0.630-1.338
	Education (high)	0.149	.547	1.161	0.714-1.886
	Self-reported health	0.610	.003	1.841	1.237-2.741
Step 7					
Sexual satisfaction	Sex (male)	−0.567	.001	0.567	0.408-0.788
	Cohabitation	0.416	.023	1.516	1.058-2.171
	Age ≥81	0.626	.006	1.870	1.192-2.933
	Self-reported health	0.626	.002	1.871	1.263-2.771

OR = odds ratio; CI = confidence interval.

### Self-Reported Health and Functional Activity

Self-reported health is a composite variable that includes 5 domains: mobility, hygiene, main activity, pain, and depression. Here, the sums of the variables were recoded as either “good health” or “poor health,” with the latter classification used for all participants who answered reported any impairment of the 5 domains.

Personal activities of daily living (PADL) capacity was measured using the rating scale from the Older Americans Resources and Services schedule, which is modified from the original scale of Lawton and Brody.^18^ The scale includes 14 items concerning the physical dimension of daily activities, with questions regarding whether the subject can eat, dress, care for their personal appearance, walk, get in and out of bed, take a bath alone without trouble, and get to the toilet in time. The index resulted in a total score ranging from 0 to 12 points, where 0 points indicated “independent” and 1–12 points symbolized “dependent”.

### Statistical Analysis

Descriptive statistical analyses were conducted to obtain an overview of the data. Backward regression analyses were performed in a logistic regression model to determine odds ratios for independent variables associated with sexually activity and satisfaction. Only first and last step are presented in Tables 3 and 4. For each odds ratio (OR), we also present the 95% confidence interval (CI). Crosstabs and the chi-square test were used to detect significant differences between groups based on sex and age. A *P*-value of <0.05 was considered significant. Analyses were compiled using the IBM SPSS Statistics v.25 for Windows by a professional statistician.

### Main Outcome Measures

Sexual activity and sexual satisfaction.

## Results

Table 1 summarizes the participant's self-stated sexual activity in accordance with demographic characteristics. Of the 1680 participants (mean age 74.7), 1414 (84%) answered the question about sexual activity, and among these 1414 respondents, 654 (46%) defined themselves as sexually active. We observed a clear decline of sexual activity in relation to age. In the youngest age cohort (60–66 years of age), 387 of 529 participants (73%) reported that they were still sexually active. In this age cohort, sexual activity was reported by 199 of 250 men (80%) and 188 of 279 women (67%). In other age cohorts, sexual activity was also reported by a larger proportion of men than women. However, the percent were similar in the oldest age cohort (≥90 years of age), with sexual activity reported by 4 of 34 men (12%) and 9 of 91 women (10%).

Table 2 presents the analysis of participants who answered the question regarding their satisfaction with their sex life (1049 of the 1680 study participants; 62%). The results showed that in the youngest 2 age cohorts (60–66 years and 72–78 years of age), 25% of participants were dissatisfied with their sex life. In contrast to the results regarding sexual activity, women were more satisfied (approximately 80%) with their sex life than men in all age cohorts. Approximately 30% of men expressed dissatisfaction with their sex life across all age cohorts.

The logistic regression analysis showed that cohabiting with a partner was positively associated with being sexually active (OR 2.309). Sexual activity was also positively associated with good self-rated health (OR 1.425). Moreover, all age cohorts were negatively related with sexual activity, with the odds ratio decreasing with increasing age (Table 3).

A logistic regression model with sexual satisfaction as the dependent variable (Table 4) showed that men are more likely to be more sexual dissatisfied than women. Those who have self-rated health as good and are cohabitating with their partner had the greatest probability of being satisfied with their sex life than the opposite. The probability of being sexually satisfied was highest in the age group 81 years and older.

## Discussion

Our present findings support previous reports showing that elderly people are sexually active and value their sexuality as an important part of their lives.^19^ We found that sexual activity was high among participants of 60 to 66 years of age (73%) and then decreased with increasing age. Across all age cohorts, approximately 75% of the population reported that they were sexually satisfied. These results compare favorably with previous research, despite different age groups and study designs.^1^^,^^17^^,^^20^

We found that at all ages, men were more sexually active than women, in agreement with previous reports.^21^ It is possible that women's sexual activity decreased at a faster rate because women in this generation often live with older men, and the woman's ability to be sexually active is negatively impacted if a man's ability falters with age.^3^ Of particular interest, here we showed for the first time in a Swedish context that approximately 10% of participants aged ≥90 years reported sexual activity, regardless of sex. In the logistic regression model, factors independently associated with sexual activity included cohabitation, good self-reported health, and male sex. Sexual activity was positively influenced by cohabiting with a partner and good self-rated health which is similar findings that have been presented in previous research.^22^ A previous study found that high degree of PADL are related to higher sexual activity,^12^ but in our study, one surprising thing was that low PADL did not have an impact on sexual activity for men, which indicates that regardless of PADL capacity, there appears to be an ability to be sexually active.

Our results also showed that older people, both men and women, were generally satisfied with their sex life. Although the men in this study were more sexually active than women, the women reported a higher degree of being sexually satisfied than men. One explanation why they are less sexually active may be that women in connection with menopause experience vulvovaginal symptoms that lead to difficulties in being sexually active.^23^ While the explanation for being more satisfied is that women have a different view of the meaning of sexual satisfaction and feel satisfied even if they are not sexually active. Studies also have reported that in men coital intercourse does not represent a crucial step to remain sexually active.^24^ Across all age cohorts, about every third man was dissatisfied with their sex life. This could be due to a number of male stereotypes that directly or indirectly affect the related interpersonal factors. It may be that as men get older, they fail to properly adjust their self-image in accordance with their increased age. This could mean that they continue to hold themselves to ideals more appropriate to their younger selves, and cannot live up to these ambitious goals, leading to dissatisfaction with their sex life. Similar results have been noted among men who have undergone treatment for erectile dysfunction.^25^ In addition to the difference between sexes, our results also showed that people who rated their health as well tended to be more satisfied with their sex life, and that the oldest age group seemed to be the most satisfied.

Although our present findings generally showed high sexual activity and satisfaction at advanced ages, there was a group of older people who do not experience sexual satisfaction. This must be studied further to obtain more comprehensive knowledge of sexual health among older people. It is likely that the elderly population of the future will differ from those included in this study. Those in the 2 oldest age groups in the present study were born in Sweden between 1910 and 1930 and grew up during a time when sexuality was not discussed and was considered primarily for reproduction rather than enjoyment. Future elderly populations will have grown up with a more liberal view of sexuality. In addition, the entry of birth control pills into the Swedish market in 1965 made it possible for women and men to enjoy their sexuality to a greater extent with reduced anxieties about pregnancy.

The present data are cross-sectional, and the results can only be interpreted as associations and cohort findings for the studied age groups. Further studies with longitudinal results are needed to draw conclusions on factors causing declines in sexual activity and satisfaction with age, and whether these findings are stable between generations. However, we think our present results can be generalized to that extent that sexual activity is present and of importance among older people, which is something that should be considered by health care professionals who work with older patients. Greater knowledge and awareness of older persons’ sexuality can increase the opportunity to talk about sexual health among older people in the health care setting.^15^^,^^26^

### Limitations of the Study

Strengths of the present study include its population-based design with relatively large sample (n = 1680); however, the study also has several flaws and the results should be interpreted with caution. For obvious reasons, limited data are available for individuals who are aged ≥90 years. Although home visits were possible, people who were very sick declined participation. In addition, the survey was not translated in other languages; therefore, people who could not read or speak Swedish could not participate in the study. The results remain limited to Swedes living in medium sized communities and may not be applicable to other populations. The use of the data from the 2001-2010 timeframe may represent a bias. The yes answers from the sexual activity question were combined, which after merging gave 2 equal groups. This can be seen as a bias but because the purpose was only to find out if the participant had been active or not, this was considered acceptable. The same applies to the other 2 self-assessed questions. There were a quite high number of no respondents to sexual activity (n = 365) who, one might think, would have naturally failed then to respond to a sexual satisfaction question as well. Finally, sexual activity was not defined in detail in our study, leaving participants to decide for themselves what they considered to be sexually active. It is possible that some participants assumed that the question about sexual activity referred only to sexual intercourse. This question is therefore examined in an interview study.^24^

## Conclusions

Our present results showed that sexual activity is present throughout life, even among the oldest age groups. Sexual activity was more common among men than women across all age groups, although this difference was less pronounced within the oldest age cohort. Among both men and women aged ≥90 years, approximately 10% were still sexually active. Across all age groups, women were more satisfied with their sex lives than men, and approximately one in 3 men reported dissatisfaction with his sex life. Positively rated health was importantly associated with sexual activity and also had a positive impact on sexual satisfaction. Overall, the present findings confirm that it is important that health professional take sexuality into account during caring encounters with older persons.

## Statement of authorship

Magnus Stentagg: Writing - Original Draft, Formal Analysis; Magnus Stentagg, Lisa Skär, Johan Sanmartin Berglund, Terese Lindberg: Conceptualization, Methodology, Investigation, Resources, Writing - Review & Editing, Funding Acquisition; Magnus Stentagg, Johan Sanmartin Berglund, Terese Lindberg: Writing - Original Draft, Formal Analysis, Project Administration; Magnus Stentagg, Lisa Skär, Johan Sanmartin Berglund, Terese Lindberg: Conceptualization, Resources, Writing - Review & Editing; Magnus Stentagg, Johan Sanmartin Berglund, Lisa Skär; Terese Lindberg: Conceptualization, Resources, Writing - Review & Editing; Magnus Stentagg, Johan Sanmartin Berglund, Lisa Skär, Terese Lindberg: Conceptualization, Resources, Writing - Review & Editing.
